# Determination of the Optimal Mixture of Wall Materials for the Encapsulation of Cacao Pod Polyphenols by Spray Drying Using Response Surface Methodology

**DOI:** 10.3390/molecules31213897

**Published:** 2026-10-28

**Authors:** Liliana Esther Sotelo-Coronado, Vincenzo Disca, Armando Alvis-Bermúdez

**Affiliations:** 1 Research Group in Commerce, Industry and Tourism (GICIT), Servicio Nacional de Aprendizaje (SENA), Montería 230002, Córdoba, Colombia; 2 Grupo de Investigaciones en Propiedades y Procesos Alimentarios (GIPPAL), Food Engineering Program, Universidad de Córdoba, Montería 230002, Córdoba, Colombia; aalvisbermudez@correo.unicordoba.edu.co; 3 Department of Pharmaceutical Sciences, Università degli Studi del Piemonte Orientale “A. Avogadro”, Largo Donegani 2, 28100 Novara, Italy; vincenzo.disca@uniupo.it

**Keywords:** encapsulation, polyphenols, cacao pod husk, cacao mucilage, response surface methodology, spray drying

## Abstract

Cacao pod husk (CPH) and cacao mucilage (CM) are abundant underutilized by-products with potential as sources and carriers of phenolic compounds. This study evaluated wall-material mixtures for the spray-drying microencapsulation of a polyphenol-rich cacao pod husk extract using an I-optimal mixture design and response surface methodology. Cacao mucilage solids, inulin, polydextrose, and sodium alginate were investigated. Process yield was the only response adequately described by the fitted model and was therefore used as the model-based response for formulation selection, whereas polyphenol recovery, encapsulation efficiency, total phenolic content, moisture content, and antioxidant capacity were evaluated experimentally. The selected formulation contained 20% CM solids, 0.65% inulin, and 79.35% polydextrose, without alginate. Experimental validation yielded a process yield of 79.93%, polyphenol recovery of 72.45%, and encapsulation efficiency of 87.95%. The resulting powder contained 4.13 mg GAE/g total polyphenols and showed an antioxidant capacity of 7.94 µg TE/g, moisture content of 3.21%, and water activity of 0.225. Microcapsules were predominantly spherical (D50 = 7.66 µm). DSC showed a Tg of 58.29 °C and additional transitions but did not establish a definitive thermal-stability limit. The findings support cacao by-products as functional spray-dried powders with low initial moisture and water activity in a circular-economy approach.

## 1. Introduction

Circular economy is recognized as an ideal model for transitioning toward sustainable production systems and mitigating the environmental impact associated with agribusiness [1]. Indeed, the recovery of residual biomass has become a priority strategy to reduce losses, close material cycles, and promote resource efficiency [2,3].

Agri-food waste can reach up to 50% in sectors such as fruit and vegetable production, underscoring the need for more sustainable production models based on the valorization of by-products rich in bioactive compounds such as polyphenols, flavonoids, carotenoids, and pectins. These compounds are widely demanded by the food, pharmaceutical, and cosmetic industries and, within the framework of the circular economy, cacao production represents a typical example of agri-food chains that generate large amounts of residual biomass very rich in valuable bioactive compounds. According to FAOSTAT (2026) [4] approximately 5.6 million tons of fermented dry cacao beans were reported in 2023. However, this figure corresponds only to the marketable fraction of the fruit, representing approximately 23.2% of the total cacao biomass [5]. Based on these data, global whole cacao production is estimated to have reached 24.13 million tons, of which 78.8% corresponds to underutilized by-products, including pod husks (67% of the fruit, approximately 16.37 million tons), mucilage (9% of the fruit, approximately 2.20 million tons), and bean shells (2% of the fruit, approximately 0.48 million tons). The valorization of this residual biomass would mitigate the environmental impact associated with the cacao production chain while representing a strategic opportunity for the development of functional ingredients and high-value-added products [6].

The reported data indicate that approximately 80% of the cacao fruit is discarded, generating a significant environmental impact and leading to the underutilization of materials with high contents of dietary fiber, lignin, cellulose, proteins, and secondary metabolites of technological interest [7]. These residues also represent an important source of antioxidants, such as theobromine, catechin, and epicatechin, which have recognized roles in the prevention of chronic diseases and in nutraceutical applications [6].

Several studies have demonstrated the potential of cacao by-products as functional resources. For example, Nguyen et al., (2025) [8] reported that the incorporation of CPH as a partial substitute for mango pulp in jam formulations increased phenolic content by 3.0–3.9 times and enhanced antioxidant activity by up to 1.5-fold, while also improving compound retention during storage. These findings highlight the capacity of cacao by-products to serve as a source of functional ingredients and sustainable applications, consolidating cacao as a key component within circular economy and biotransformation strategies [6].

Phenolic compounds represent one of the most relevant bioactive fractions in food science due to their well-documented antioxidant capacity and their contribution to the prevention of chronic non-communicable diseases [9]. Indeed, this class of compounds exhibit antioxidant and anti-inflammatory properties and may modulate metabolism, immunity, gut microbiota, and oxidative stress. Their intake has been associated with reduced risk of cardiovascular, metabolic, neurodegenerative, and cancer-related diseases. However, their application is limited by poor solubility, instability, low bioavailability, and sensitivity to light, oxygen, and temperature [10,11].

In this context, the valorization of agro-industrial cacao by-products through advanced extraction, bioconversion, and microencapsulation technologies offers a promising strategy to obtain functional ingredients with improved stability, protection, and bioactivity. Simultaneously, it reduces the environmental impact of the production chain and supports sustainable, zero-waste models [6,7,12,13]. Emerging technologies, particularly microencapsulation, have gained strategic relevance due to their ability to improve the stability, protection, and controlled release of bioactive compounds during digestion, thereby strengthening their functional effectiveness [14].

The intrinsic instability of bioactive compounds has encouraged the use of functionalization technologies, particularly nano- and microencapsulation, to protect them, improve stability during processing and storage, and enable targeted release with greater biological efficiency. Several studies have reported that cacao cob, peel, and other fruit by-products are rich in polyphenols, highlighting their potential as valuable resources within circular economy models [6,15,16]. Accordingly, the valorization of cacao residues offers an opportunity to recover high-value compounds and develop more stable, safe, and bioactive nutraceutical ingredients through suitable encapsulation strategies.

Microencapsulation by spray drying is one of the most widely applied techniques due to its ability to immobilize thermosensitive bioactive compounds, thereby reducing their deterioration caused by temperature, light, oxygen, and pH, while ensuring controlled release and efficient handling of functional ingredients. The final performance of the encapsulated system is strongly influenced by processing parameters such as inlet air temperature, feed viscosity, and matrix composition, which govern the formation of the protective film and the retention of polyphenols during atomization. Optimal processing conditions promote rapid droplet solidification and minimize the loss of functional compounds, resulting in microcapsules with adequate solubility, structural stability, and high functional performance [17].

The literature highlights that process efficiency depends critically on the selection of wall materials, whose functionality determines the ability to form protective matrices, control hygroscopicity, modulate water activity, and preserve the integrity of the core compounds during processing and storage. In particular, polymeric combinations such as maltodextrin and gum arabic have been shown to enhance emulsion stability, optimize encapsulation efficiency, and extend the shelf life of functional powders [18]. Furthermore, parameters such as particle size, morphology, encapsulation efficiency, and release profile have been reported to depend directly on the type of coating material and crosslinking mechanisms, which in turn condition the bioaccessibility and stability of the encapsulated compounds, reinforcing the critical role of materials engineering in the development of functional microcapsules [19,20].

In accordance with the above, spray drying has established itself as a technology of high interest within the scientific community [21,22]. Among the most widely explored strategies for preserving and valorizing functional compounds, spray drying stands out due to its versatility and scalability.

In this context, the present study aimed to evaluate the influence of wall-material composition on the spray-drying microencapsulation of polyphenols extracted from cacao pod husk using an I-optimal mixture design and Response Surface Methodology (RSM). Process yield was evaluated as the model-based response for formulation selection, whereas polyphenol recovery, encapsulation efficiency, total phenolic content, antioxidant capacity, moisture content, and selected physicochemical properties of the resulting microcapsules were experimentally characterized.

## 2. Results and Discussion

### 2.1. Optimization of the Wall-Material Mixture and Process Performance

The experimental results were obtained using a four-component I-optimal mixture design combined with Response Surface Methodology (RSM) to optimize the proportions of the wall materials used for the microencapsulation of the CPH hydroethanolic extract. Among the evaluated components, dried cacao mucilage was considered, as it constitutes a by-product of the cacao agroindustry with high functional potential and added value. Its incorporation represents an innovative alternative for waste valorization within circular economy approaches [6].

The remaining wall materials, namely inulin, polydextrose, and alginate, were selected based on their well-documented structural stability and capacity to retain bioactive compounds, properties that make them effective systems for microencapsulation by spray drying and related techniques [23,24].

Table 1 presents the 27 mixture-design runs, while Table 2 and Table 3 summarize the experimental responses and analysis of variance, respectively. As shown in Table 2, process yield ranged from 64.21% (run 27) to 85.99% (run 6), while polyphenol recovery varied between 58.16% (run 25) and 83.54% (run 6). Encapsulation efficiency exhibited the greatest variability, with values ranging from 53.70% (run 8) to 89.05% (run 23). Total polyphenol content fluctuated between 3.73 mg GAE/g (run 21) and 4.89 mg GAE/g (run 5). Moisture content ranged from 2.18% (run 8) to 4.21% (run 25), whereas antioxidant capacity determined by the DPPH assay showed comparatively lower variability, with values between 6.08 µg TE/g dry basis (run 20) and 9.90 µg TE/g dry basis (run 5).

The experimental data presented in Table 2 were subsequently input into Design-Expert^®^ software version 13 (Stat-Ease, Inc., Minneapolis, MN, USA). to model and analyze the influence of mixture components on the evaluated response variables, enabling the identification of significant interactions and response behavior trends.

The statistical analysis indicated that process yield was the only response adequately described by a statistically significant quadratic Scheffé mixture model (F = 10.36, *p* < 0.0001). The fitted model showed an R^2^ of 0.8457 and an adjusted R^2^ of 0.7641, indicating that a substantial proportion of the variability in process yield was explained after accounting for model complexity. Furthermore, the low coefficient of variation (C.V. = 3.54%) indicated satisfactory experimental precision, while the non-significant lack-of-fit test (*p* = 0.3823) supported the adequacy of the model within the investigated mixture region. Collectively, these statistical parameters indicate an adequate fit of the model to the experimental data. Process yield is directly related to product recovery and is determined by the amount of powder recovered after the drying or encapsulation process [25]. The fitted quadratic mixture model for process yield is expressed as follows:

Yield = 344.27A + 77.52B + 76.43C + 28,276.89D − 286.76AB − 310.17AC − 31,587.80AD + 3.71BC − 29,279.25BD − 28,903.44CD

where A is cacao mucilage, B is inulin, C is polydextrose, and D is alginate.

The large numerical magnitude of the coefficient associated with sodium alginate (D) should be interpreted within the constrained nature of the mixture design rather than as an independent effect on process yield. Sodium alginate was evaluated over a considerably narrower experimental range (0–2%) than the other mixture components; moreover, the D term should be interpreted jointly with the AD, BD, and CD interaction terms, which exhibited coefficients of comparable magnitude and opposite sign. Therefore, the magnitude of this coefficient reflects the parameterization of the model within the experimental mixture region rather than a physical increase of such magnitude in process yield.

Graphical analysis of the response surfaces at different alginate levels (0%, 1%, and 2%) showed that the highest yield was achieved in the absence of alginate (Figure 1). As the alginate proportion increased, a progressive decrease in yield was observed. This behavior is consistent with reports indicating that alginate increases feed emulsion viscosity during spray drying, thereby reducing atomization efficiency and overall process performance. In encapsulation processes, alginate concentrations generally do not exceed 3% of the total formulation [21,26]. Nevertheless, its application should be evaluated on a case-by-case basis, as successful spray-drying encapsulation of different *Lactobacillus* probiotics using alginate has also been reported [27].

Among the significant effects identified, both linear mixture terms and several second-order interactions were observed, particularly the interactions A–B (cacao mucilage–inulin), A–C (cacao mucilage–polydextrose), A–D (cacao mucilage–alginate), B–D (inulin–alginate), and C–D (polydextrose–alginate). Residual analysis showed no outliers, and the normal distribution of standardized residuals supported the validity and robustness of the proposed model (Table 3).

In contrast, for the polyphenol recovery and encapsulation efficiency responses, it was not possible to obtain models with satisfactory predictive capacity, suggesting that these variables do not exhibit a statistically significant dependence on the proportions of the encapsulating materials within the evaluated experimental range.

Among the candidate formulations generated within the experimental region, the formulation containing 20% cacao mucilage solids, 0.65% inulin, and 79.35% polydextrose, with no alginate, was selected for experimental validation (Table 4). Selection was primarily based on maximizing cacao mucilage incorporation, in accordance with the valorization objective of the study, while maintaining a high predicted process yield of 80.48%, the response adequately described by the fitted mixture model. Because satisfactory predictive models were not obtained for polyphenol recovery and encapsulation efficiency, these responses were not used as model-based criteria for the final selection and were instead assessed experimentally during validation.

Formulation selected for experimental validation. Process yield values represent predictions from the fitted quadratic Scheffé mixture model.

Within the context of microencapsulation, inulin has been widely described as a wall material with multiple technological and functional advantages. Inulin exhibits film-forming properties that facilitate the formation of a protective barrier around the core material, thereby protecting encapsulated compounds and enhancing their stability [28,29]. In addition, inulin is resistant to hydrolysis by digestive enzymes in the human gastrointestinal tract, enabling the controlled release of bioactive compounds in the small intestine [28]. Beyond its technological role, inulin also acts as a prebiotic, as it is a non-digestible polysaccharide fermented by colonic microbiota, stimulating the growth of beneficial bacteria such as *Bifidobacterium* spp., which are associated with human health benefits [29].

The results obtained in the present study regarding the performance of polydextrose and inulin are consistent with those reported by Tamtürk et al., (2023) [30], who demonstrated that wall material composition significantly influences chlorophyll encapsulation performance by spray drying, achieving values of up to 78.4%. These authors also confirmed the contribution of inulin to maximizing encapsulation efficiency. Similarly, Sapei et al., (2025) [31] showed that inulin enhances encapsulation efficiency and reinforces antioxidant properties when used as a wall material in oil encapsulation systems. In line with these findings, Corrêa-Filho et al., (2022) [28] reported that the combination of polydextrose and inulin as carrier materials enabled the encapsulation of betacyanins into nanoparticles with high stability and controlled gastrointestinal release profiles, characterized by higher release in the gastric phase and delayed release in subsequent phases. In a related study, the same authors further demonstrated that nanoparticle-based encapsulation of sensitive pigments effectively mitigated instability caused by factors such as temperature, light, and oxygen, thereby expanding their potential applicability in the food industry.

The strategy of prioritizing mucilage utilization responds not only to technical objectives but also to circular economy and sustainability criteria. Cacao honey, also known as cacao mucilage, is characterized by its high carbohydrate and total sugar contents, acidic nature, and low ash content (approximately 0.2%). It exhibits a low lipid fraction (<3.54%) and provides essential minerals such as calcium, magnesium, and phosphorus. Dietary fiber content is generally low (<1%); however, higher values (up to approximately 16.89%) have been reported in some cases, reflecting variability associated with origin and processing conditions. Reported values of total soluble solids range between 14.03 °Brix and 19.6 °Brix [32,33]. These compositional characteristics justify the interest in its utilization, while also explaining the technological challenges it presents, mainly derived from its high sugar and pectin contents.

Nevertheless, the high hygroscopicity of cacao mucilage limits its inclusion at proportions higher than 20%, as excessive incorporation may promote moisture absorption and lead to particle agglomeration, thereby reducing the physical stability of the powder. This behavior is consistent with that reported for sugar-rich materials, which typically exhibit low glass transition temperatures (Tg) and high solubility, resulting in yield losses during spray drying [34]. Despite these limitations, the results of the present study demonstrate that stable microcapsules containing a substantial proportion of mucilage can be obtained without compromising process performance or antioxidant functionality.

In this context, Guirlanda, (2022) [33] conducted an exploratory study on the spray drying of cacao honey powder, evaluating different carrier proportions, including maltodextrin, methocel, and whey protein isolate (WPI). The reported results support the potential of this by-product as an alternative wall material. More broadly, the literature recognizes that cacao by-products, including pod husk, mucilage, and bean shell, represent valuable resources for food and pharmaceutical applications due to their content of bioactive compounds with functional and antioxidant properties [6]. Their valorization not only contributes to sustainability by transforming waste into value-added inputs but also improves income opportunities for smallholder producers and stimulates innovation within the food industry [35]. Furthermore, the use of these residues as “green” wall materials aligns with clean-label trends and sustainable by-product management, although their technological feasibility and scalability still require further investigation [30].

Further research is required to evaluate formulations containing mucilage proportions higher than 20%, accompanied by strategies aimed at mitigating its hygroscopic limitations. Potential approaches include the use of co-encapsulants with a greater capacity to form stable matrices, structural modification of polysaccharides, or the application of secondary coatings to reduce moisture absorption. Such studies would enable a deeper understanding of the influence of mucilage on critical variables such as process yield, encapsulation efficiency, polyphenol retention, microcapsule morphology, and antioxidant capacity.

### 2.2. Physicochemical and Functional Properties of the Optimized Microcapsules

The selected formulation was experimentally validated through three independent spray-drying runs (*n* = 3), with each analytical determination performed in triplicate; results are expressed as mean ± standard deviation. The experimentally determined process yield was 79.93 ± 4.63%, compared with the model-predicted value of 80.48%, corresponding to a relative validation error of 0.68%. This small deviation, which remained within the experimental variability observed among the validation runs, supports the adequacy of the fitted model for describing process yield within the investigated mixture region. Polyphenol recovery and encapsulation efficiency were 72.45 ± 4.72% and 87.95 ± 6.26%, respectively. Their corresponding relative deviations from the numerical values initially generated during formulation selection (71.90% and 89.05%) were 0.76% and 1.24%, respectively. Because satisfactory predictive models were not obtained for these two responses, these deviations are reported descriptively and are not interpreted as predictive-model validation errors. The experimentally determined process yield and polyphenol recovery were within the ranges reported for spray-dried encapsulated systems [36,37], while the high encapsulation efficiency indicates effective retention of phenolic compounds within the wall-material matrix. The combined use of inulin, polydextrose, and cacao mucilage solids may have contributed to the formation of the encapsulating matrix, consistent with the technological roles reported for these carrier materials in spray-drying systems [30,37].

The resulting powder showed a moisture content of 3.21 ± 0.28% and a water activity (a\(_w\)) of 0.22 ± 0.02. The low moisture content was below the 5% level commonly associated with reduced susceptibility of spray-dried powders to moisture-related phenomena such as hygroscopicity, agglomeration, and structural collapse [38]. These values characterize the initial physicochemical condition of the freshly produced powder; however, they should not be interpreted as evidence of long-term storage stability, which would require dedicated real-time or accelerated stability studies.

Regarding the functional characteristics of the validated formulation, the powder exhibited a total phenolic content of 4.13 ± 0.12 mg GAE/g dry basis and an antioxidant capacity of 7.94 ± 0.64 µg TE/g dry basis, demonstrating that measurable phenolic compounds and antioxidant activity were retained after spray drying. These results support the functional potential of the resulting microencapsulated powder without attributing the observed values exclusively to the protective effect of the wall materials.

### 2.3. Particle Morphology and Elemental Composition

Morphological analysis of the sample corresponding to the optimal formulation was performed by FE-SEM (Figure 2).

The observed microstructure exhibited particles with a predominantly spherical morphology, which is consistent with and expected for structures generated by spray drying. During this process, atomized droplets tend to contract uniformly as water evaporates, resulting in smooth surfaces and regular contours [38]. However, variations in particle size were observed, with the presence of smaller primary particles tending to agglomerate around larger ones, suggesting phenomena associated with partial coalescence or post-drying surface adhesion.

This aggregation pattern is characteristic of matrices with a high content of soluble solids, such as the combination employed in this study, in which cacao mucilage, inulin, and polydextrose contribute to increased viscosity and promote intermolecular interactions that influence agglomerate formation. Similar behavior has been reported by (Dieplinger et al. 2023 [36]), who showed that the use of low-molecular-weight saccharides affected the particle size distribution of encapsulated systems. Likewise, Siemons et al., (2020) [37] demonstrated that the use of different dextrose equivalents led to coalescence effects, which became more pronounced as the dextrose equivalent increased in maltodextrin. Consequently, the use of maltodextrin-type carriers in combination with cacao mucilage favors the aggregation behavior observed in this study.

Regarding surface uniformity, most particles exhibited a continuous and homogeneous texture, with no evidence of structural cracks or collapse, indicating that the drying conditions were adequate to preserve the integrity of the encapsulating wall. Nevertheless, some microcapsules presented surface microdepressions and bulges, which may be associated with temperature and humidity gradients during atomization, as well as differences in solubility and mobility among the mixture components. Such phenomena have been attributed to internal process variations related to feed solution homogeneity, viscosity fluctuations, compositional differences, thermal gradients, and water evaporation dynamics during capsule formation [37,39].

Complementary elemental composition analysis by EDS revealed that the optimal sample consisted predominantly of oxygen (65.9%) and carbon (33.8%), consistent with the organic nature of the wall materials [20]. Trace amounts of potassium (0.3%), attributable to the mineral fraction of cacao mucilage, were also detected [40]. No detectable levels of magnesium or phosphorus were observed. This elemental composition confirms the predominance of organic constituents with low inorganic content, which is consistent with the intended technological function of the encapsulating system and its potential applicability in food matrices.

Overall, the morphological and elemental characterization suggests that the obtained microcapsules exhibit an adequate surface structure to ensure effective physical protection of the core material, with high structural integrity and mechanical stability. Spherical morphology, the marked absence of fractures, and a unimodal particle-size distribution covering a relatively broad size range are key factors for optimizing dispersion in solid or liquid matrices and for controlling the release of bioactive compounds in food applications [41,42].

### 2.4. Particle-Size Distribution

The particle-size distribution showed D10, D50, and D90 values of 2.53 ± 0.10 µm, 7.66 ± 0.19 µm, and 19.48 ± 0.31 µm, respectively (Figure 3). The corresponding span, calculated as (D90 − D10)/D50, was 2.21, indicating appreciable particle-size dispersion despite the unimodal character of the distribution. This size distribution is consistent with the heterogeneous particle dimensions commonly observed in spray-dried encapsulated systems [43].

The formation of amorphous solid dispersions during spray drying depends not only on solvent evaporation kinetics but also on interactions between the bioactive compounds, carrier materials, and solvent, as well as on the physicochemical state of the feed solution. Formulation parameters, such as feed concentration and solvent type, together with process conditions, including spray rate and drying gas flow, ultimately determine the final particle structure. Consequently, both the encapsulation technique and the properties of the wall and core materials exert a decisive influence on particle morphology. Smaller and more uniformly distributed particles have been shown to exhibit enhanced physical stability, reduced aggregation tendencies, and improved storage performance. In addition, their higher specific surface area favors solubility, biocompatibility, and controlled release of encapsulated compounds [44,45].

Overall, the results suggest that the developed formulation constitutes an efficient microencapsulation system for polyphenols derived from cacao residues, with relevant implications for the valorization of agro-industrial by-products and the development of high-value-added functional foods.

### 2.5. Thermal Properties

Differential scanning calorimetry (DSC) analysis of the microencapsulated system composed of cacao mucilage solids, inulin, and polydextrose revealed several thermal transitions (Figure 4). A glass transition temperature (Tg) of 58.29 °C was observed, corresponding to the transition of the amorphous matrix from a glassy state to a state of increased molecular mobility. A low-intensity endothermic event was detected below 85.21 °C (ΔH = 0.8343 J/g), which may be associated with the release of residual or bound moisture. A broader and more intense endothermic event was also observed between 121.96 and 130.91 °C (ΔH = 4.342 J/g).

However, this event should be interpreted as a thermal transition of the encapsulating matrix rather than as conclusive evidence of thermal degradation. Because DSC records changes in heat flow but does not directly measure mass loss, degradation of the matrix or loss of encapsulated compounds cannot be confirmed solely from DSC data. Complementary thermogravimetric analysis would be required to establish the occurrence and onset of thermal decomposition. Accordingly, no definitive thermal-stability limit was assigned to the microencapsulated system based solely on the DSC thermogram shown in Figure 4.

### 2.6. Study Limitations

Although inulin was incorporated as a wall material with recognized prebiotic functionality, the gastrointestinal behavior of the optimized microparticles was not evaluated in the present study. Consequently, the current results do not allow conclusions regarding digestive stability, gastrointestinal release, or the bioaccessibility of the encapsulated polyphenols. These aspects should be addressed in future studies using standardized in vitro gastrointestinal digestion models.

## 3. Materials and Methods

### 3.1. Samples

Samples consisted of cacao genotypes cultivated by smallholder farmers and producer associations in the Upper Sinú region, specifically in the municipalities of Valencia and Tierralta (Córdoba, Colombia). A non-probabilistic convenience sampling strategy was applied, selecting Los Campanos farm, located in the village of San Clemente, municipality of Tierralta. Samples were collected from different lots within the farm. The raw materials included mucilage, CPH, obtained from an equal mixture (1:1:1) of local genotypes FEAR 5, ICS 39, and FSV 41, selected based on their regional representativeness.

### 3.2. Preparation of Polyphenol Extracts

CPH were cut into pieces measuring approximately 1 cm × 1 cm with a thickness of 3 mm using a Fuller Machinery^®^ food processor (Fuller Machinery Ltd.a., Bogotá, Colombia) [46]. The material was then dried at 50 °C for 48 h until a moisture content below 5% (*w*/*v*) was reached. Subsequently, the dried material was ground using a MGM-810 laboratory grinder (Industrias Quantik S.A.S., Quimbaya, Quindío, Colombia) to obtain particle sizes between 300 and 500 µm. Polyphenol extraction was performed by ultrasound-assisted extraction, following the methodology reported by Sotelo et al. (2015) [46] and Berkani et al. (2020) [43]. The extraction was carried out using a ultrasonic bath, 60 Hz (BIOBASE Group, Jinan, Shandong, China) for 60 min at 50 °C with a hydroethanolic solvent (50:50, *v*/*v*; Merck KGaA, Darmstadt, Germany). The resulting extract was centrifuged at 4000 rpm for 10 min and filtered through No. 1 filter paper.

The resulting hydroethanolic extract exhibited a total phenolic content (TPC) of 37.53 ± 2.13 mg GAE/g on a dry basis, as determined by the Folin–Ciocalteu method. For the preparation of the spray-drying formulations, extract incorporation was standardized to provide a constant phenolic load of 406.5 mg GAE in a final feed volume of 500 mL. The total solids concentration of the feed solution was adjusted to 11.25% (*w*/*v*) for all experimental formulations. The pH of the hydroethanolic extract and feed solutions was not determined during the original experiments.

### 3.3. Obtaining Cacao Mucilage

Cacao mucilage was collected as leachate during the first three hours of the fermentation process. Immediately after collection, the mucilage was pasteurized at 75 °C for 15 min and subsequently stored at −20 °C until use.

For formulation preparation, the mucilage was not subjected to any prior concentration, freeze–drying, or drying treatment. Its moisture content was determined gravimetrically at 105 °C to constant weight according to AOAC Method 943.06 (AOAC, 2012 [47], using a Memmert UN110 plus forced-convection oven Memmert GmbH + Co. KG, Schwabach, Germany). The total solids fraction of the mucilage was subsequently calculated by difference. The amount of liquid mucilage incorporated into each formulation was established on a dry-matter basis so that the contribution of mucilage solids corresponded to the proportion specified in the experimental design. The pasteurized mucilage was incorporated directly, in its liquid state, into the spray-drying feed solution. The intrinsic water content of the mucilage was included in the overall aqueous-phase balance of the formulation, and the system was adjusted to maintain a total feed-solids concentration of 11.25%. Accordingly, the term “cacao mucilage solids” used in the experimental design refers to the dry-matter fraction contributed by the mucilage to the wall-material mixture rather than to a previously dehydrated ingredient.

Cacao mucilage was selected as a wall material because of its pectin and sugar content [48,49]. The formulation was complemented with inulin, polydextrose, and sodium alginate (Sigma-Aldrich, Merck KGaA, Darmstadt, Hesse, Germany), selected based on their physicochemical properties, stability, and ability to contribute to the retention and protection of bioactive compounds during spray-drying microencapsulation and related encapsulation processes [23,30,50].

### 3.4. Total Polyphenol Content Analysis

Total polyphenol content (TPC) was determined using the Folin–Ciocalteu method, using analytical-grade Folin–Ciocalteu reagent (Merck KGaA, Darmstadt, Germany) as described by [7]. Absorbance was measured at 760 nm using a BIOBASE^®^ FlexA-200 microplate spectrophotometer (Hangzhou Allsheng Instruments Co., Ltd., Hangzhou, China). Gallic acid was used as the calibration standard, and results were expressed as mg gallic acid equivalents per gram of dry matter (mg GAE/g).

### 3.5. Determination of DPPH Radical Scavenging Activity

DPPH radical scavenging activity was determined according to the methodology described by Disca et al. (2024) [7]. Briefly, appropriately diluted samples were analyzed using the 2,2-diphenyl-1-picrylhydrazyl radical (DPPH•). An aliquot of 700 µL of the diluted sample was mixed with 700 µL of a 100 µmol/L DPPH• solution prepared in methanol (Merck KGaA, Darmstadt, Germany). For the control, 700 µL of methanol was mixed with 700 µL of the DPPH• solution.

The reaction mixtures were vigorously mixed and incubated for 20 min at room temperature in the dark to minimize photo-induced degradation of the DPPH radical. After incubation, absorbance was measured at 515 nm using a Shimadzu UV-1900 UV–Vis spectrophotometer (Shimadzu Corporation, Tokyo, Japan).

Antioxidant capacity was quantified by comparison with a Trolox calibration curve, using Trolox (6-hydroxy-2,5,7,8-tetramethylchroman-2-carboxylic acid) as the reference antioxidant. All determinations were performed in triplicate, and the results were expressed as Trolox equivalents per gram of dry matter (µg TE/g, dry basis).

### 3.6. Experimental Design

During the evaluation of the wall-material mixtures, process yield, polyphenol recovery, and encapsulation efficiency were considered as primary technological responses, while total phenolic content, antioxidant capacity, and moisture content were evaluated as complementary quality attributes of the resulting powders. Among these responses, process yield was the only variable adequately described by a statistically significant fitted mixture model and was therefore used as the model-based response for numerical optimization. Polyphenol recovery and encapsulation efficiency were retained as experimental performance indicators and were subsequently assessed during validation of the selected formulation.

A four-component I-optimal mixture design was used to evaluate the effects of cacao mucilage solids, inulin, polydextrose, and sodium alginate on the experimental responses. For process yield, the final fitted model corresponded to a quadratic Scheffé mixture model, comprising the linear mixture terms and binary interaction terms among the four components. The selected formulation was determined by maximizing process yield together with cacao mucilage incorporation within the investigated experimental region, whereas polyphenol recovery and encapsulation efficiency were not used as predictive criteria in the final model-based optimization.

*y* = *a*_1_*x*_1_ + *a*_2_*x*_2_ + *a*_3_*x*_3_ + *a*_4_*x*_4_ + *a*_12_*x*_1_*x*_2_ + *a*_13_*x*_1_*x*_3_ + *a*_14_*x*_1_*x*_4_ + *a*_23_*x*_2_*x*_3_ + *a*_24_*x*_2_*x*_4_ + *a*_34_*x*_3_*x*_4_

The runs of the experimental design are shown in Table 1. During experimentation, fixed operating variables were established to ensure process reproducibility. The total polyphenol content in the feed solution was 406.5 mg of gallic acid equivalents (GAE), with a total solids concentration of 11.25%. The spray drying process was conducted at an inlet air temperature of 150 °C and a feed flow rate of 6 mL/min, using a total feed volume of 500 mL. These operating conditions were taken as reference values for the optimization study Grassia et al., (2021) [51]. The aspirator rate was maintained at 80%, and the atomization air pressure at the nozzle was set to 1 bar.

### 3.7. Preparation of the Spray-Drying Feed Dispersion and Spray-Drying Conditions

For each experimental run, the amounts of wall materials were established according to the proportions defined by the mixture design and expressed on the basis of the solids contributed by each component. Inulin, polydextrose, and sodium alginate were previously dispersed in portions of distilled water and allowed to hydrate for 4 h before preparation of the final feed dispersion. Pasteurized cacao mucilage was incorporated directly in its liquid form. Its moisture content was previously determined and the total-solids fraction was calculated by difference; this value was used to determine the amount of liquid mucilage required to provide the proportion of cacao mucilage solids specified for each experimental run. Thus, the term cacao mucilage solids used in the experimental design refers to the dry-matter contribution of the mucilage and does not indicate the use of previously dried mucilage. The intrinsic water supplied by the liquid mucilage was included in the overall aqueous-phase balance of the formulation.

The hydroethanolic polyphenol extract was used without prior ethanol removal or subsequent reconstitution. For each formulation, 52.1 mL of the extract obtained using water:ethanol (50:50, *v*/*v*) was incorporated into a final feed volume of 500 mL. Based on the nominal composition of the extraction solvent, the maximum theoretical ethanol concentration in the final feed dispersion was approximately 5.21% (*v*/*v*). The prehydrated wall materials, liquid cacao mucilage, and hydroethanolic extract were combined, and the final volume was adjusted to 500 mL with distilled water while maintaining a total solids concentration of 11.25% (*w*/*v*) and a phenolic load of 406.5 mg gallic acid equivalents (GAE) per feed batch.

Immediately before spray drying, the final feed dispersion was homogenized using an Ultra-Turrax homogenizer (IKA-Werke GmbH & Co. KG, Staufen, Germany) at 11,000 rpm for 10 min at room temperature. The feed dispersion was neither filtered nor degassed after homogenization and was immediately transferred to the spray dryer. Continuous mechanical stirring was maintained in the feed reservoir during processing to minimize sedimentation or phase separation and to maintain a uniform feed. Spray drying was performed using a Büchi B-290^®^ Mini Spray Dryer (Büchi Labortechnik AG, Flawil, Switzerland) at an inlet air temperature of 150 °C, a feed flow rate of 6 mL/min, an aspirator setting of 80%, and an atomization pressure of 1 bar.

### 3.8. Evaluation of Encapsulation Efficiency, Polyphenol Recovery, and Process Yield

Total phenolic content (TPC) and surface phenolic content (SPC) were determined following the methodology described by Grassia et al. (2019) [52]. For TPC determination, 100 mg of microcapsules was dispersed in 1 mL of an ethanol–acetic acid–water mixture (50:8:42, *v*/*v*/*v*). The dispersion was vortexed for 1 min and subsequently sonicated twice for 20 min. The resulting extract was centrifuged at 6000 rpm for 10 min and filtered prior to analysis. For SPC determination, 100 mg of microcapsules was dispersed in 1 mL of an ethanol–methanol mixture (1:1, *v*/*v*), vortexed for 1 min at room temperature, and filtered through a 0.45 µm Millipore^®^ membrane filter. In both cases, phenolic compounds were quantified using the Folin–Ciocalteu method.

Encapsulation efficiency (EE), polyphenol recovery (R), and process yield (Y) were calculated according to Equations (1)–(3), respectively:

Encapsulation Efficiency (EE)

(1) $$(EE)=\frac{TPC-SPC\;}{TPC}\times 100$$

where TPC is the total phenolic content of the microcapsules and SPC is the surface phenolic content, both expressed on the same analytical basis. Thus, EE represents the proportion of phenolic compounds retained within the encapsulating matrix relative to the total phenolic content measured in the powder.

Polyphenol recovery (R):

(2) $$R\;(\%)=\frac{TPCmicroparticules}{TPC,\;\;feed\;}\times \;\;100$$

where TPC microcapsules is the total phenolic content determined in the spray-dried microcapsules and TPC feed is the total phenolic content of the corresponding feed before spray drying, both expressed on the same analytical basis. Polyphenol recovery therefore represents the percentage of the initial phenolic content retained in the microcapsules after spray drying.

Process yield (Y):

(3) $$Y\;(\%)=\frac{\;MP,\;powder}{Mdry\;solids,\;feed}\times \;100$$

where m,powder is the mass of spray-dried powder recovered after processing (g), and mdry, solids,feed is the total mass of dry solids initially present in the feed before spray drying (g). Based on a total feed-solids concentration of 11.25% (*w*/*v*) and a final feed volume of 500 mL, the theoretical initial dry-solids mass corresponded to 56.25 g per experimental batch.

The selected formulation was experimentally validated through three independent spray-drying runs (*n* = 3), with each analytical determination performed in triplicate. Validation results were expressed as mean ± standard deviation. For process yield, model validation was assessed by comparing the model-predicted value with the experimental mean, and the relative validation error was calculated as the absolute difference between the experimental and predicted values divided by the predicted value and multiplied by 100.

### 3.9. Particle Morphology and Size

Microparticle morphology was analyzed by field-emission scanning electron microscopy (FESEM) coupled with energy-dispersive X-ray spectroscopy (EDS) for elemental identification. The analysis was conducted following ASTM E1508-12a [53], Standard Guide for Quantitative Analysis by Energy-Dispersive Spectroscopy. A Thermo Scientific™ Apreo™ 2 S field-emission scanning electron microscope (Thermo Fisher Scientific, Brno, Czech Republic) equipped with a Thermo Fisher Scientific EDS analyzer (ANAX-30P-B UltraDry™, 30 mm^2^ detector) was used. Measurements were performed under controlled environmental conditions (18–20 °C and 68.0% relative humidity). Particle size distribution was determined by laser diffraction using a Mastersizer 3000 (Malvern Panalytical Ltd., Malvern, Worcestershire, UK) coupled with the Aero S dry dispersion unit.

### 3.10. Moisture Content and Water Activity

The moisture content of the powders was determined by gravimetric analysis at 105 °C until constant weight, according to AOAC Official Method 943.06 AOAC (2012) [47]. Water activity (a_w) of the microparticles was measured following the NMKL 168 standard, *Water activity—Instrumental determination*.

### 3.11. Differential Scanning Calorimetry

The thermal behavior of the microencapsulated powders was evaluated using a Mettler Toledo^®^ differential scanning calorimeter (DSC, DSC1 model Mettler-Toledo International Inc., Greifensee, Switzerland). Sample preparation was performed under controlled ambient conditions (25 °C and 30% relative humidity). Approximately 15 mg of each microencapsulation sample were sealed in aluminum pans and heated from 0 to 140 °C at a heating rate of 5 °C/min under a constant nitrogen flow [54].

## 4. Conclusions

The application of an I-optimal mixture design combined with Response Surface Methodology enabled the identification of process yield as the only response adequately described by the fitted statistical model. The optimized wall-material system, comprising cacao mucilage solids, inulin, and polydextrose, resulted in microcapsules with a process yield of 79.93%, a polyphenol recovery of 72.45%, and an encapsulation efficiency of 87.95%. Within the experimental region evaluated, the inclusion of alginate was associated with reduced process performance, including significant interaction effects with other mixture components. In contrast, prioritizing cacao mucilage within the optimized formulation supported the valorization of this agro-industrial by-product while maintaining satisfactory technological performance of the encapsulation system.

Regarding functional preservation, the optimized microencapsulated powder exhibited a total phenolic content of 4.13 mg GAE/g on a dry basis and an antioxidant capacity of 7.94 µg TE/g dry basis, indicating retention of phenolic functionality following spray drying. The low moisture content (3.21%) and water activity (0.225) further reflected limited water availability in the freshly produced powder, a physicochemical condition associated with reduced susceptibility to moisture-related phenomena such as hygroscopicity and agglomeration. However, these initial physicochemical attributes should not be interpreted as evidence of long-term stability; therefore, the retention of phenolic compounds and antioxidant capacity during storage remains to be confirmed through dedicated real-time or accelerated stability studies.

Morphological characterization revealed predominantly spherical microcapsules with continuous surfaces and no evidence of structural collapse. Furthermore, particle-size analysis showed a unimodal distribution, with a median particle diameter (D50) of 7.66 µm and a span of 2.21, indicating appreciable size dispersion among the obtained microparticles.

Finally, the use of cacao agro-industrial by-products demonstrated its relevance as a sustainability-driven strategy. Cacao mucilage and pod husks were successfully transformed into inputs with high functional value and technological applicability. The incorporation of these residues as sources of bioactive compounds and alternative encapsulation materials contributes to the development of functional foods with potential health benefits, while simultaneously promoting circular economy principles and the integral valorization of the cacao production chain.

## Figures and Tables

**Figure 1:**
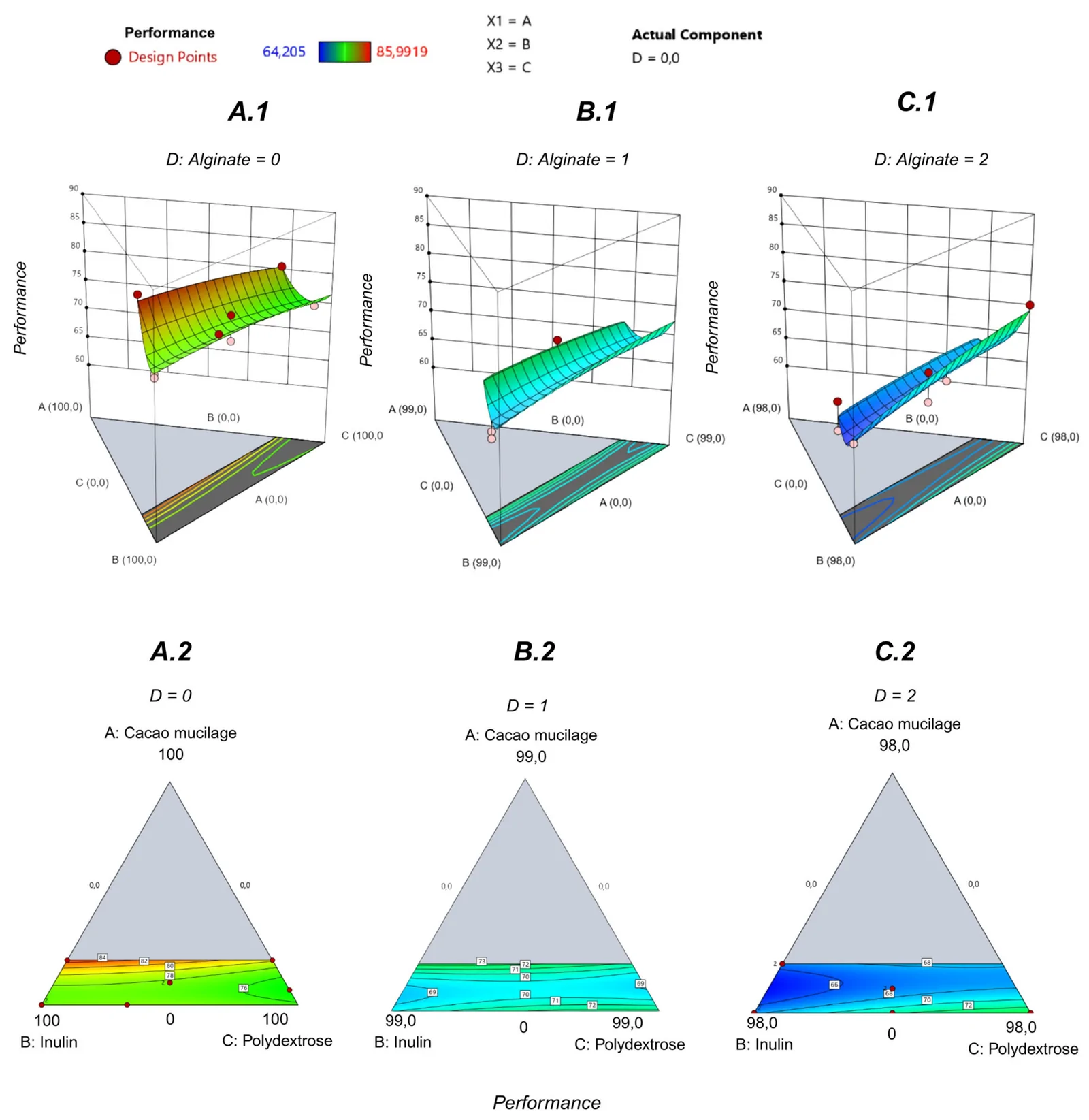
Effect of sodium alginate content on the microencapsulation optimization response surfaces: (**A.1**,**A.2**) 0% alginate; (**B.1**,**B.2**) 1% alginate; and (**C.1**,**C.2**) 2% alginate.

**Figure 2:**
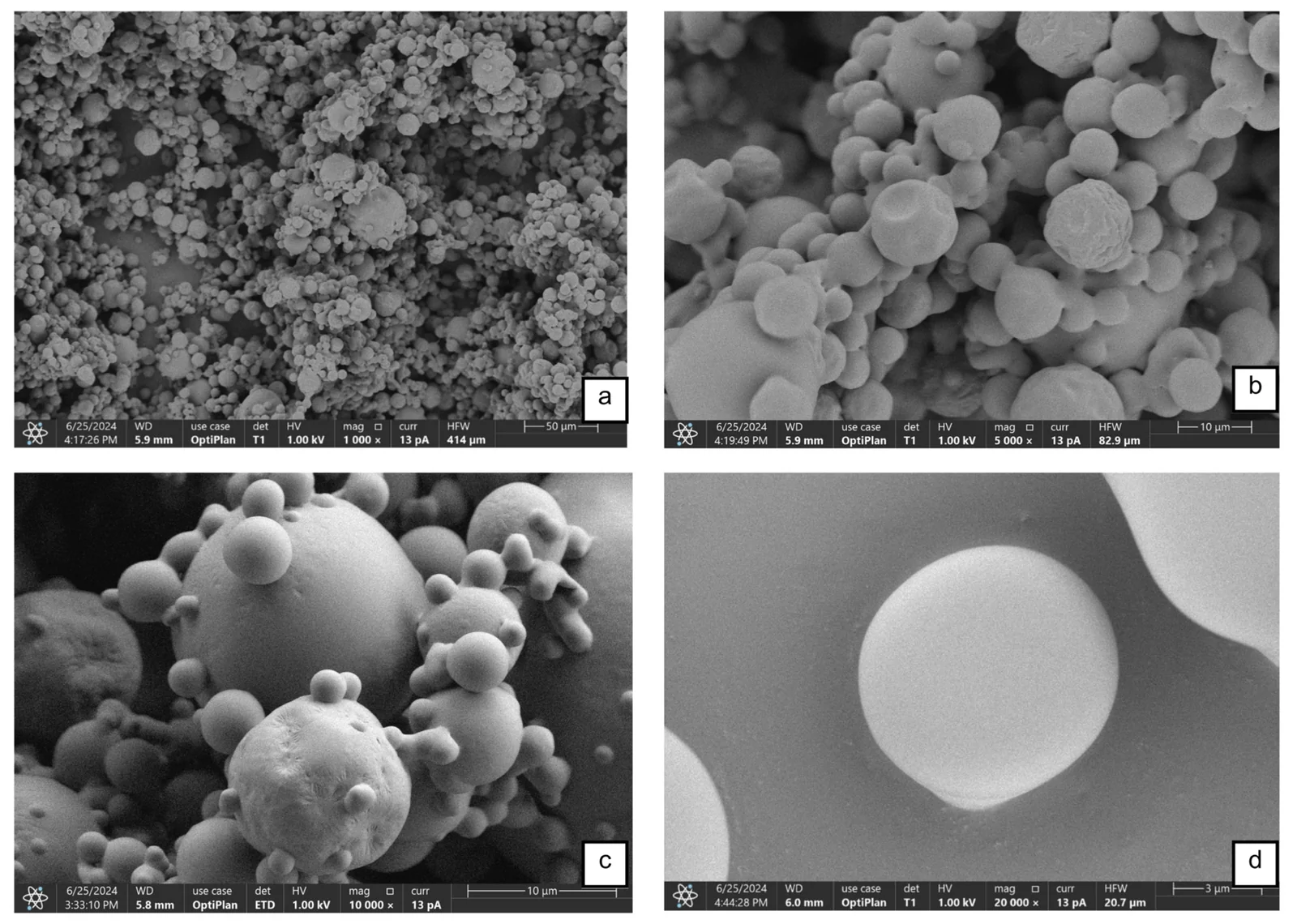
Morphology of the optimized formulation observed by field-emission scanning electron microscopy (FE-SEM) at (**a**) 1000×, (**b**) 5000×, (**c**) 10,000×, and (**d**) 20,000× magnification.

**Figure 3:**
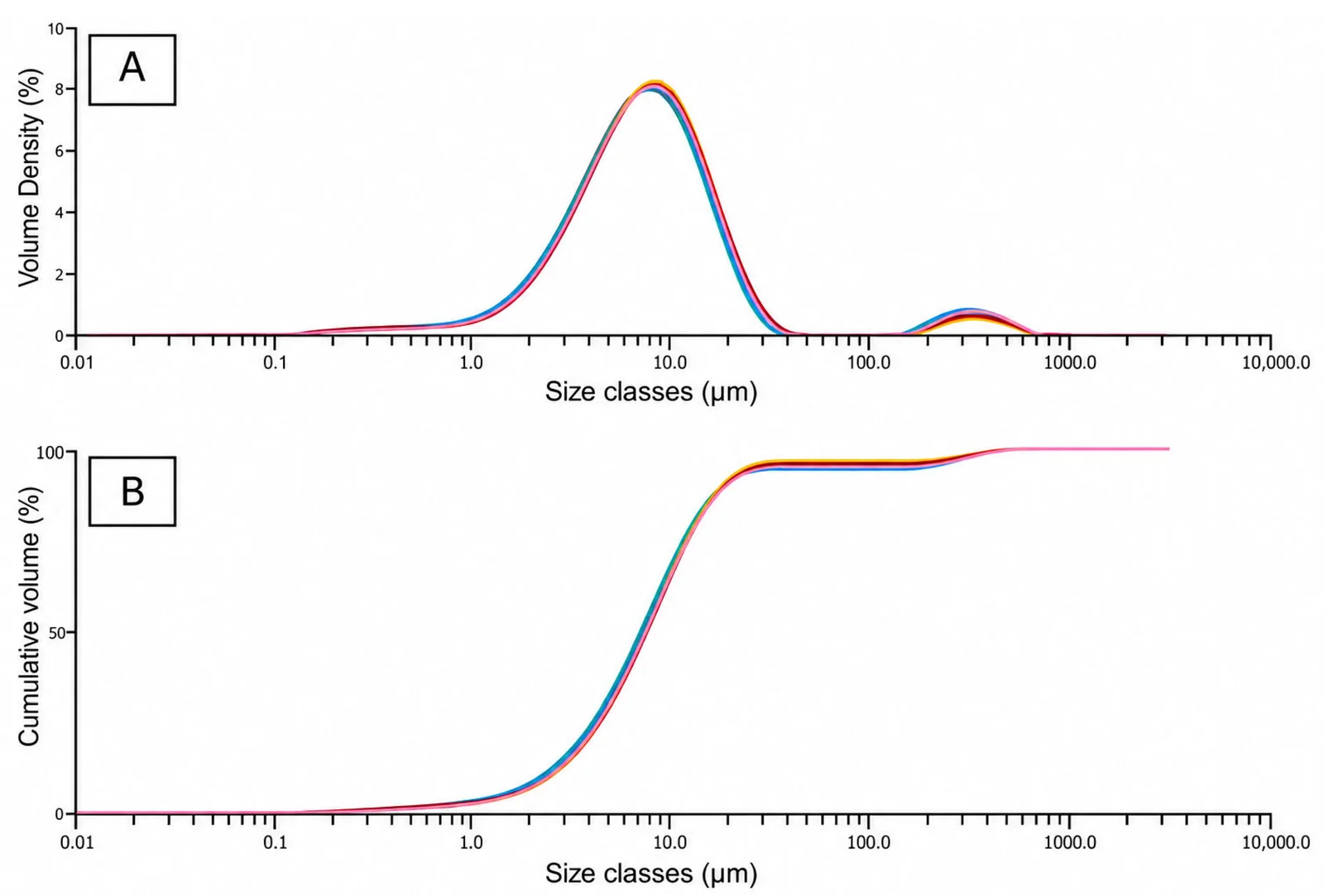
Particle-size distributions of the optimized formulation measured using a Mastersizer 3000: (**A**) differential volume distribution and (**B**) cumulative volume distribution.

**Figure 4:**
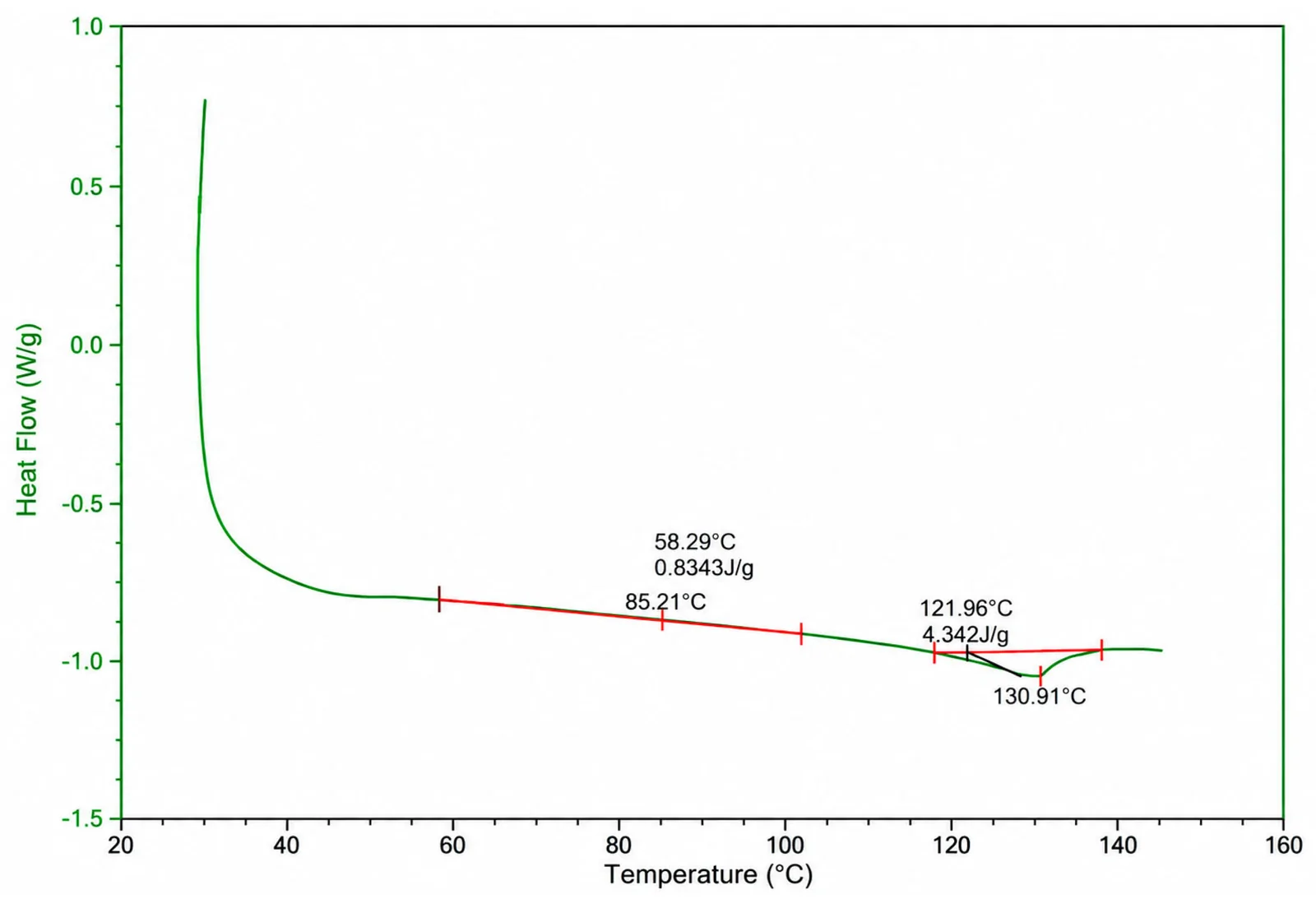
Differential scanning calorimetry thermogram of the optimized formulation.

**Table 1:** Mixture-design runs generated using Design-Expert software version 13 (Stat-Ease, Inc., Minneapolis, MN, USA).

	Component 1	Component 2	Component 3	Component 4
Run	A: Cacao Mucilage Solids (%)	B: Inulin (%)	C: Polydextrose (%)	D: Alginate (%)
1	5	22.3	72.3	0.5
2	10	45	45	0
3	10	45	45	0
4	6.7	0	93.3	0
5	0	98	0	2
6	20	80	0	0
7	10	44	44	2
8	20	78	0	2
9	0	0	98	2
10	20	39.5	39.5	1
11	20	0	78.7	1.3
12	0	66.7	33.3	0
13	5	72.3	22.3	0.5
14	15	61.3	22.3	1.5
15	20	39.5	39.5	1
16	0	100	0	0
17	15	22.3	61.3	1.5
18	0	33.3	66	0.7
19	10	89	0	1
20	0	33.3	66	0.7
21	0	100	0	0
22	20	78	0	2
23	20	0	80	0
24	0	98	0	2
25	0	49	49	2
26	10	89	0	1
27	10	44	44	2

**Table 2:** Experimental responses for the 27 mixture-design runs.

	Response 1	Response 2	Response 3	Response 4	Response 5	Response 6
Run	Process Yield (%)	Recovery (%)	Encapsulation Efficiency (%)	Total Polyphenols (mg GAE/g)	Moisture (%)	Antioxidant Capacity (µg TE/g)
1	75.73	73.16	66.76	4.41	2.68	8.68
2	78.56	70.59	64.93	4.33	2.39	8.83
3	74.31	64.35	69.64	4.17	2.79	9.08
4	74.27	58.12	87.96	3.77	2.67	8.88
5	70.34	75.54	54.24	4.89	2.45	9.90
6	85.99	83.54	60.12	4.57	3.55	8.61
7	69.30	60.37	66.58	4.19	2.25	8.84
8	69.30	65.56	53.70	4.56	2.19	8.51
9	74.91	71.62	76.36	4.60	2.20	8.83
10	73.80	72.81	57.03	4.75	2.86	8.91
11	69.29	62.25	69.49	4.33	2.31	8.70
12	78.86	73.52	56.53	4.49	3.04	7.21
13	76.54	75.28	60.40	4.73	3.44	8.88
14	70.63	68.86	60.48	4.69	2.72	9.35
15	71.13	70.07	65.77	4.74	3.72	8.64
16	76.84	72.43	59.43	4.54	3.64	8.86
17	68.24	67.56	64.93	4.76	2.74	7.06
18	71.16	63.66	65.80	4.31	3.72	7.88
19	65.60	62.05	82.25	4.55	2.35	8.05
20	73.17	62.91	75.42	4.14	2.29	6.08
21	77.40	60.06	76.79	3.74	3.16	7.86
22	64.48	63.31	83.83	4.73	3.09	6.75
23	80.48	78.71	89.05	4.71	2.52	7.64
24	66.72	59.29	69.63	4.28	3.28	8.66
25	69.07	58.58	86.63	4.08	4.21	8.89
26	66.76	65.13	72.61	4.70	2.85	9.73
27	64.21	59.07	77.93	4.65	3.76	9.06

**Table 3:** Analysis of variance for the fitted process-yield model.

Source of Variation	Sum of Squares	df	Mean Square	F-Value	*p*-Value
Model	614.25	9	68.25	10.36	<0.0001
Linear Mixing	429.60	3	143.20	21.73	<0.0001
AB	40.47	1	40.47	6.14	0.0240
AC	45.87	1	45.87	6.96	0.0173
AD	45.19	1	45.19	6.86	0.0180
BC	2.57	1	2.57	0.3896	0.5408
BD	38.18	1	38.18	5.79	0.0277
CD	37.32	1	37.32	5.66	0.0293
Residue	112.04	17	6.59		
Lack of fit	65.45	9	7.27	1.25	0.3823
Pure Error	46.59	8	5.82		
Total	726.29	26			
R^2^	0.8457				

**Table 4:** Candidate wall-material formulations and model-predicted process yield generated during formulation selection.

Response/Component	1 *	2	3	4	5
Cacao mucilage solids (%)	20.00	16.67	16.22	18.38	16.67
Inulin (%)	0.65	0.00	3.73	8.1624	83.33
Polydextrose (%)	79.35	83.33	80.05	73.00	0.00
Alginate (%)	0.00	0.00	0.00	0.00	0.00
Predicted process yield (%)	80.48	77.99	77.92	83.52	72.91

* Selected solution in the optimization process.

## Data Availability

The data supporting the findings of this study are included in the article and are available from the corresponding author upon reasonable request.
